# Management of renal cell carcinoma with solitary metastasis

**DOI:** 10.1186/1477-7819-3-48

**Published:** 2005-07-20

**Authors:** Yuvaraja B Thyavihally, Umesh Mahantshetty, Ravichand S Chamarajanagar, Srinivas G Raibhattanavar, Hemant B Tongaonkar

**Affiliations:** 1 Department of Genito-urinary oncology, Tata Memorial Hospital, Mumbai, India; 2 Radiation Oncology, Tata Memorial Hospital, Mumbai, India; 3 Preventive oncology, Tata Memorial Hospital, Mumbai, India

## Abstract

**Background:**

Distant metastasis are common in Renal cell carcinoma (RCC) nearly one forth of the patients have metastasis at presentation while another 50% develop metastasis during the follow-up. A small percentage of these are solitary metastasis. We describe survival after surgical excision or radiotherapy of solitary metastatic lesion from renal cell carcinoma

**Patients and methods:**

Between 1988–2001, 43 patients with solitary metastasis to different sites from renal cell carcinoma underwent either surgical excision or radiotherapy were analyzed. The solitary nature of the lesions was confirmed by investigations. All patients have had radical nephrectomy for the primary lesion. Survival analysis was carried out by Kaplan Meier Method.

**Results:**

All solitary metastatic lesions were treated with intent of cure either by excision or radiotherapy. Of these, 13 patients had solitary metastasis at the time of presentation in whom 3-year overall median survival was 26 months. The survival of those who developed solitary metastases during follow-up after nephrectomy for primary was 45 months. The patients with long interval between diagnosis and development of metastasis, early stage and low grade of the primary tumor had better prognosis.

**Conclusion:**

Complete resection of either synchronous or metachronous solitary metastases from renal cell carcinoma is justified and can contribute to a long-term survival in this select group of patients.

## Background

Nearly 20–25% of patients with renal cell carcinoma (RCC) have distant metastasis at presentation. Another 50% develop metastasis or local recurrence during follow-up after the treatment of the primary [1]. RCC can recur at any time after nephrectomy and usually metastasizes via venous and lymphatic routes. Frequent sites of metastasis include the lung parenchyma (50% to 60%), [2] bone (30% to 40%), [3] liver (30% to 40%), and brain (5%) [4]. Other rare sites of metastasis include pancreas [5,6], adrenal gland [7,8], parotid gland [9], maxilla, pharynx etc. Even with early-stage disease, late metastases can occur after complete resection. However, after the development of metastasis the prognosis is often poor, as non-operative modalities for advanced renal carcinoma have failed to improve survival significantly. The average survival of metastatic RCC is about 4 months and only 10% of these survive for one year. Chemotherapy, hormonal therapy, and radiotherapy have generally proved ineffective for primary lesion or metastatic deposits, but an improved survival is seen with the use of immunotherapy with either interleukins or interferon [10,11]. There is a small subset of patients where solitary metastasis is present either at the time of presentation or develops during follow-up after nephrectomy these patients have a better survival. An autopsy series showed that 8 to 11% of the patients with metachronous metastases have a solitary lesion [12]. With the introduction of immunotherapy or immunochemotherapy for advanced renal cell carcinoma, survival has improved for a subset of patients. Surgical resection of the renal tumor and solitary metastases if present is still the treatment of choice for these lesions [13]. By modern perioperative management, even extended resections of metastatic lesions can be performed with limited morbidity and mortality [14]. The present study reports the result of retrospective analysis of the records of 43 patients treated for solitary synchronous or metachronous metastases from renal cell carcinoma.

### Patients and methods

Between 1988 and 2001, 1863 patients of RCC were treated of these 43 cases had solitary metastasis to different sites. Thirteen patients had synchronous solitary metastsis, whereas 30 patients developed solitary metastasis during follow-up after the treatment of the primary in a metachronous fashion. The location and extent of the metastatic disease was evaluated by various diagnostic methods, which include chest radiograph, isotope bone scan, liver function tests, and serum alkaline phophatase. All patients underwent either ultrasound (USG) or computerized tomography (CT) of abdomen to rule out local recurrence. The patients with lymph node metastasis were excluded from the analysis. An attempt was made to obtain histological proof of the metastatic lesion either by fine needle aspiration cytology (FNAC) or USG/CT guided biopsy. The analysis was performed separately for patients with synchronous and metachronous metastatic lesions. Survival analysis was calculated by using Kaplan-Meier test and survival was compared using Log-rank test.

## Results

### Synchronous group

**Figure 1:**
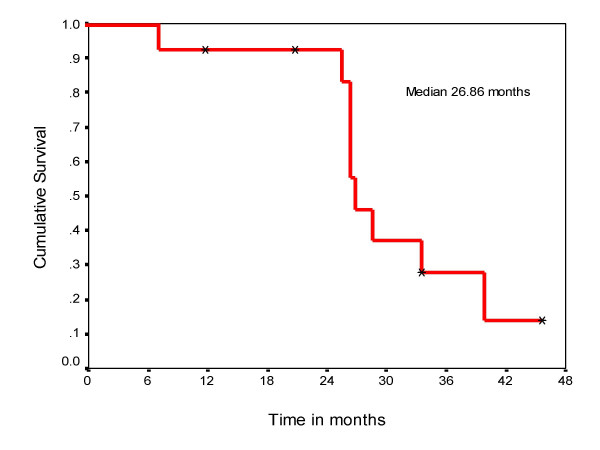
Overall survival in patients with synchronous solitary metastasis.

Thirteen patients with synchronous solitary metastatic disease included 10 males and 3 females. The age of the patients ranged from 38 to 69 years with mean of 57 years. The site of solitary metastases were bone in 6, lung in 3, liver in 2, and 1 each in brain and opposite adrenal gland (Table 1). All patients underwent radical nephrectomy for the primary in the same sitting except in one patient with brain metastasis, which was irradiated. One patient with bone metastasis had positive surgical margin and received radiotherapy. The median disease free survival was 25 months and the median overall survival was 26 months with 3-year survival being 27% and none of the patients surviving 5 years (Figure 1). Five patients who could afford received interferon alpha treatment for relapse to multiple sites. Due to small number of patients a subgroup analysis was not carried out for these patients.

**Table 1:** Patient and tumor characteristics- synchronous solitary metastasis

Total No. of patients	13
Age	38–69 years (mean = 57)
Sex	Male-10, Female-3
Site of metastasis	Bone-6, Lung-3, Liver-2, Brain-1, Opposite adrenal-1
Pathological stage of primary	pT1–3, pT2–6, pT3–4
Fuhrman's Grading	Grade I-1, Grade II-4, Grade III-5, Grade IV-3

### Metachronous group

**Figure 2:**
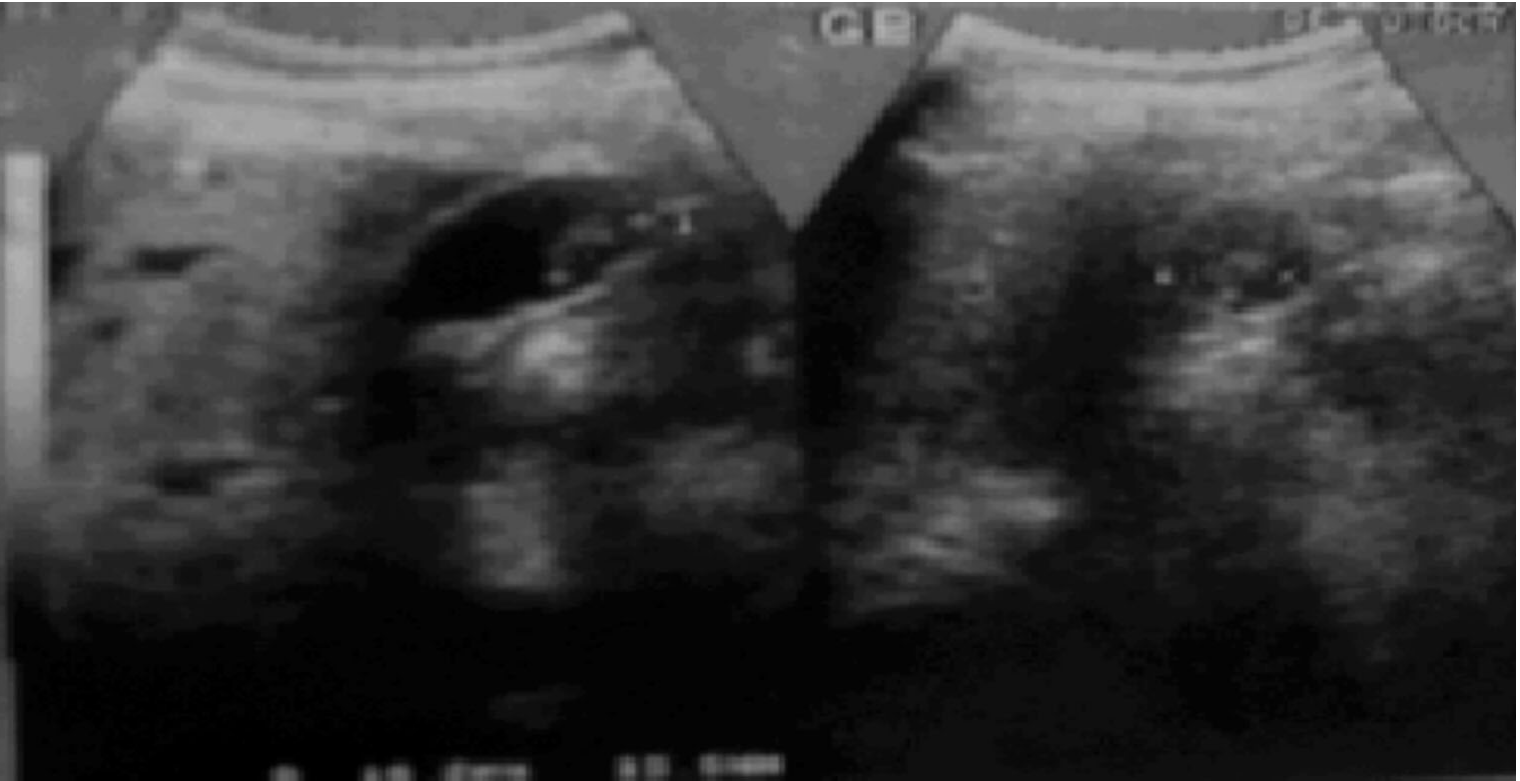
Ultrasonography showing solitary metachronous metastasis to gall bladder.

Thirty patients developed metachronous solitary lesion during follow-up after definitive treatment of the primary renal cell carcinoma of the kidney. The age of the patients ranged from 34 to 75 with the mean of 54 years. There were 21 males and 9 female patients. The metastatic interval varied between 3 to 33 months with mean of 16 months. The distribution of site of solitary metastasis was bone in 13, lung in 6, liver in 3, brain in 2, and one each in parotid, maxilla, pharyngeal wall, soft tissue shoulder, opposite adrenal and gall bladder (Figure 2). Eleven patients with bone metastases underwent excision of the lesion and other 2 patients received definitive radiotherapy. All patients with lung and liver metastases underwent surgical excision. One patient with brain metastasis received radiotherapy and other one had surgical excision of the lesion. The patient with parotid lesion underwent parotidectomy and the one with metastasis in soft tissue shoulder had forequarter amputation. Adrenalectomy and cholecystectomy was done for adrenal and gall bladder metastasis respectively. Primary tumor stage was pT1in 9, pT2 in 13, and pT3 in 8 patients. The distribution of histological types and the nuclear grading is shown in the table 2.

**Table 2:** Patient and tumor characteristics- metachronous solitary metastasis

Total No. of patients	30
Age	34–75 Years (Mean 54)
Sex	Male-21, Female-9
Metastatic interval	3–33 months (Mean 16)
Site of metastasis	Bone-13, Lung-6, Liver-3, Brain-2, Parotid-1, Maxilla-1, Pharyngeal wall-1, Gall bladder-1, Opposite adrenal-1, Soft tissue shoulder-1.
Histology of primary	Conventional Clear cell carcinoma -25 Papillary-4 Chromophobe -1
Pathological stage of primary	pT1–9, pT2–13, pT3–8
Fuhrman's Grading	Grade I-5, Grade II-12, Grade III-7, Grade IV-6

**Figure 3:**
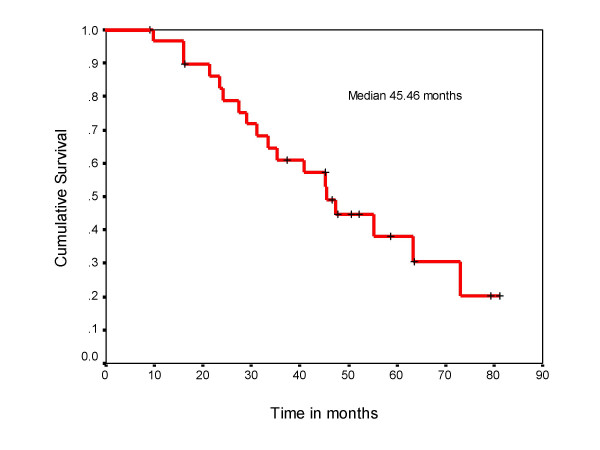
Overall survival of the patients with metachronous solitary metastasis.

**Figure 4:**
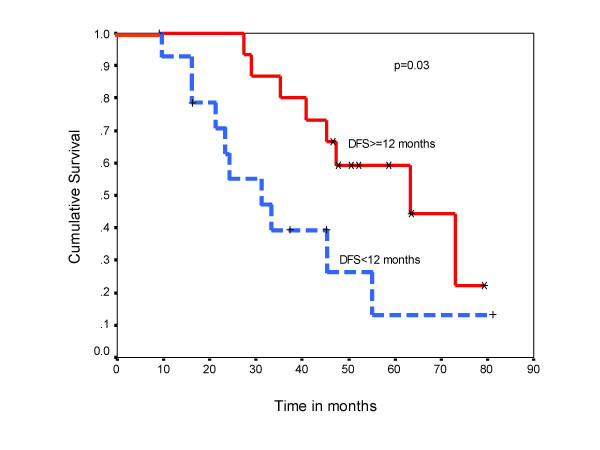
Overall survival comparing patients with metachronous solitary metastasis occurring within 12 months versus those occurring after 12 months.

Post metastatectomy overall median survival was 45 months. The 3-year survival rate was 60 % and 5-year survival was 38 % (Figure 3). The overall survival for those patients in whom solitary metastasis occurred within one year (n = 15) was 31 months compared to 63 months in those who developed metastasis after one year (n = 15) the difference was statistically significant (log rank test p = 0.03) (Figure 4). The three patients with liver metastasis and 2 with brain metastasis behaved poorly with survival of 6 to 20 months. Two patients with bone metastasis and one patient with brain metastasis received radiotherapy after excision due to positive surgical margins.

**Figure 5:**
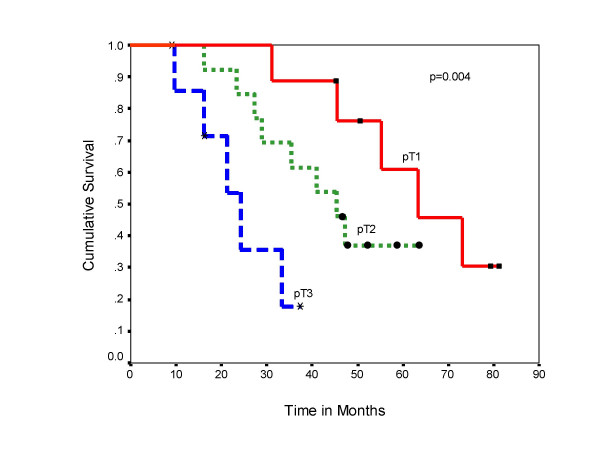
Overall survival according to pathologic stage of the primary tumor.

**Figure 6:**
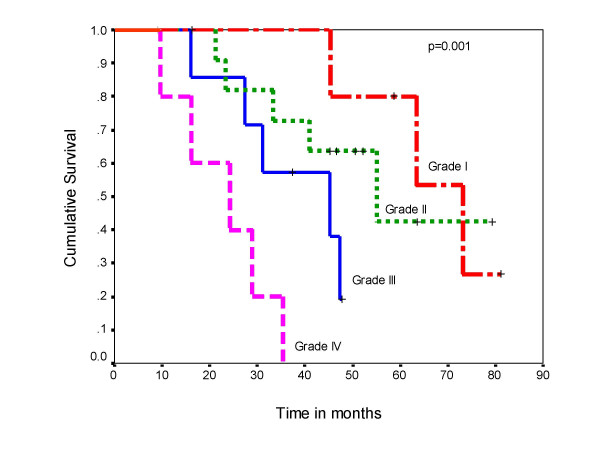
Overall survival according to Fuhrman's nuclear grading.

The overall post metastatectomy median survival was 63 months for pT1 tumor, 45 months for pT2 and 24 months for pT3 tumors and the difference in the survival was significant (log rank test p = 0.004) (Figure 5). There was also significant difference in the survival (log rank test p = 0.001) among patients with Fuhrman's grade I to grade IV disease (Figure 6). The patients with lung and bone metastasis had overall mean survival of 62 months and those with brain and liver metastasis was 22 months. Thirteen patients received immunotherapy after relapse at multiple sites following treatment of the solitary metastasis. However, the number of patients in each group was small, hence subgroup analysis could not be carried out for these patients.

## Discussion

RCC is known for its varied presentation and its propensity to metastasize by way of both venous and lymphatic routes. Ritchie and deKernion [15] found that 23% of patients with RCC present initially with metastatic disease and 25% develop metastatic disease within 5 years of nephrectomy. RCC can, however, metastasize to virtually any organ, including the thyroid, pancreas, skeletal muscle, and skin or underlying soft tissue.

Despite the early promising results with immunotherapy, a complete response occurs in less than 15% and is rarely durable, emphasizing that metastatic disease best treated with complete resection of the primary and metastatic lesions where possible. Solitary metastases are identified at the time of diagnosis of renal cell carcinoma in 2% to 4% of patients. Depending on the location of the solitary metastasis, radical nephrectomy [16] with excision of the solitary metastatic lesion has been advocated, with 20% to 30% 5-year survival rates being reported.

Barney in 1961 [17] did nephrectomy and lobectomy for lung metastasis and patient survived for 23 years. This influenced many workers to perform excision of solitary metastasis. Middleton [18] reviewed the literature and concluded that the survival after surgical excision of solitary metastatic lesions was 45% at 3-years and 34% at 5-years. Tolia and Whitmore [19], Morrow *et al *[20], and Jett *et al *[21] also reported similar 5-year survival after the treatment of solitary metastatic tumors. Skinner *et al *[22] reported a 29% 5-year survival in a series of 41 patients in whom one or two metastases were excised surgically in addition to nephrectomy. In our series, the 3 and 5-year post metastatectomy survival was 58% and 35% respectively in metachronous group of patients whereas 3-year survival was 20% in the synchronous group, the higher survival could be due to number of patients being loss to follow-up and hence censored from the survival analysis.

Middleton [18] found significant difference in survival between synchronous and metachronous solitary metastases, synchronous group being the worst. O'Dea *et al *[23] and Rafla [24] also agreed that the patients with synchronous solitary metastasis have poor survival when compared to patients who develop metastasis after removal the primary tumor. Although the site of metastasis was not significant in many reports we found patients with lung and bone metastases fared much better (median 62 months) than with liver and brain metastases (median 22 months). This could be due to the fact that not all patients with brain metastasis had undergone surgical resection, and more use of adjuvant treatment in patients with lung or bone metastasis.

Most metachronous metastases are identified in the first or second year after nephrectomy. Tally *et al *[25] Skinner *et al *[22] and Tongaonkar *et al *[26] found that survival in metachronous metastases which appeared before one year after the nephrectomy was poor (median overall survival 33 months) when compared to one in whom metastases appeared after one year (median overall survival 55 months). Our results also suggests that over all survival in patients with disease free interval less than one year was 31 months when compared to 63 months in those disease free intervals was more than one year.

We also noted significant difference in the survival among patients of different primary tumor stage and Fuhrman's nuclear grading with early stage and low grade having better survival. Thus, the groups of patients who do better with solitary metastasis excision are those with disease free interval of more than one year, low primary tumor stage, low grade and those with bone and pulmonary parenchymal metastasis. However, the effect of treatment modality and adjuvant immunotherapy can not be calculated due to smaller number of cases in the present series.

## Conclusion

A small subset of patients with a solitary metastasis from renal cell carcinoma and good general condition may benefit from nephrectomy and resection/radiotherapy of the metastatic lesion. Surgical excision can locally control the tumor, and relieve pain. Patients with disease free interval of more than one year before development of solitary metastasis, early primary tumor stage, low-grade and patients with bone and pulmonary parenchymal metastasis appear to have better survival. We believe that the relatively prolonged survival of these small cohort of patients with solitary metastatic lesions from RCC justify aggressive surgical excision involving multispecialty specialists with long-term survival, however, prospective studies are needed before this can be recommended as standard practice.

## Competing interests

The author(s) declare that they have no competing interests.

## Authors' contributions

**TY **designed the study, prepared the manuscript, and drafted the manuscript.

**MU **gave radiation details and participated in the preparation of manuscript.

**RS **participated in the design of the study and performed the statistical analysis.

**CR **participated in its design and coordination and helped to draft the manuscript.

**TH **overall monitoring of the analysis and editing of the final version for publication.

All authors read and approved the manuscript
